# Predicting major clinical events among Canadian adults with laboratory-confirmed influenza infection using the influenza severity scale

**DOI:** 10.1038/s41598-024-67931-9

**Published:** 2024-08-08

**Authors:** Henrique Pott, Jason J. LeBlanc, May ElSherif, Todd F. Hatchette, Shelly A. McNeil, Melissa K. Andrew, the Serious Outcomes Surveillance (SOS) Network of the Canadian Immunization Research Network (CIRN), Guy Boivin, Sylvie Trottier, Francisco Diaz-Mitoma, Chris Verschoor, Grant Stiver, William Bowie, Karen Green, Allison McGeer, Jennie Johnstone, Mark Loeb, Kevin Katz, Phillipe Lagacé-Wiens, Bruce Light, Anne McCarthy, Andre Poirier, Jeff Powis, David Richardson, Makeda Semret, Stephanie Smith, Geoff Taylor, Daniel Smyth, Louis Valiquette, Duncan Webster

**Affiliations:** 1 Canadian Centre for Vaccinology, Dalhousie University, Halifax, Canada; 2 Department of Medicine, Universidade Federal de São Carlos, Rod. Washington Luis, km 235, São Carlos, SP 13656-905 Brazil; 3 Department of Pathology, Dalhousie University, Halifax, Canada; 4 Department of Medicine (Infectious Diseases), Dalhousie University, Halifax, Canada; 5 Department of Medicine (Geriatrics), Dalhousie University, Halifax, Canada; 6 Centre Hospitalier Universitaire de Québec, Quebec, QC Canada; 7 Health Sciences North Research Institute, Sudbury, ON Canada; 8 University of British Columbia, Vancouver, BC Canada; 9 Mount Sinai Hospital, Toronto, ON Canada; 10 McMaster University, Hamilton, ON Canada; 11 North York General Hospital, Toronto, ON Canada; 12 St. Boniface Hospital, Winnipeg, MB Canada; 13 The Ottawa Hospital, Ottawa, ON Canada; 14 Centre Intégré Universitaire de santé et services sociaux, Quebec, QC Canada; 15 Michael Garron Hospital, Toronto, ON Canada; 16 William Osler Health System, Brampton, ON Canada; 17 McGill University, Montreal, QC Canada; 18 University of Alberta Hospital, Edmonton, AB Canada; 19 The Moncton Hospital, Moncton, NB Canada; 20 Université de Sherbrooke, Sherbrooke, QC Canada; 21 Horizon Health, Saint John, NB Canada

**Keywords:** Influenza, Risk adjustment, Major clinical events, Outcomes, Health care, Medical research, Risk factors

## Abstract

We developed and validated the Influenza Severity Scale (ISS), a standardized risk assessment for influenza, to estimate and predict the probability of major clinical events in patients with laboratory-confirmed infection. Data from the Canadian Immunization Research Network’s Serious Outcomes Surveillance Network (2011/2012–2018/2019 influenza seasons) enabled the selecting of all laboratory-confirmed influenza patients. A machine learning-based approach then identified variables, generated weighted scores, and evaluated model performance. This study included 12,954 patients with laboratory-confirmed influenza infections. The optimal scale encompassed ten variables: demographic (age and sex), health history (smoking status, chronic pulmonary disease, diabetes mellitus, and influenza vaccination status), clinical presentation (cough, sputum production, and shortness of breath), and function (need for regular support for activities of daily living). As a continuous variable, the scale had an AU-ROC of 0.73 (95% CI, 0.71–0.74). Aggregated scores classified participants into three risk categories: low (ISS < 30; 79.9% sensitivity, 51% specificity), moderate (ISS ≥ 30 but < 50; 54.5% sensitivity, 55.9% specificity), and high (ISS ≥ 50; 51.4% sensitivity, 80.5% specificity). ISS demonstrated a solid ability to identify patients with hospitalized laboratory-confirmed influenza at increased risk for Major Clinical Events, potentially impacting clinical practice and research.

## Introduction

Influenza is a respiratory viral infection that affects millions worldwide yearly. The impact of influenza can vary depending on several factors, such as the virus, the host, and contextual factors like the degree of match achieved between vaccine and circulating strains, vaccine coverage, and pre-existing population immunity^1–3^. Despite this variability, influenza remains a significant burden on people’s health worldwide, with approximately one billion cases annually, of which 3–5 million are severe and 290,000–650,000 result in influenza-related deaths^4,5^.

Although most cases have a benign course, some are at increased risk of adverse clinical outcomes, including children < 5 years, older adults, and those with a high comorbidity burden^2,6,7^. On the other hand, influenza vaccination and the timely use of antivirals have proved effective in attenuating these outcomes^2,3,8,9^ Understanding the benefits of these interventions requires a comprehensive evaluation in relation to illness severity, a gap in existing knowledge.

Influenza severity ranges from mild illness treated at home without any intervention or seen on an outpatient basis to more severe illness, including the need for ventilatory support, intensive care unit (ICU) admission, or death. A recent review revealed several popular tools employed for assessing the severity of influenza and community-acquired pneumonia, such as the Pneumonia Severity Index (PSI), CURB-65, Acute Physiology And Chronic Health Evaluation II (APACHE II), Sequential Sepsis-related Organ Failure Assessment (SOFA), and quick SOFA (qSOFA)^7^. Although these tools are used to estimate the severity of influenza, they are not specific to it.

Thus, there is an unmet need for a standardized risk assessment for influenza, particularly to characterize and estimate the probability of experiencing adverse clinical outcomes by score or a predetermined risk level and to adjust studies assessing the effect of interventions on these outcomes. In addition to being robust, this tool must be simple enough to allow its application in retrospective and prospective studies. Such a tool would enable public health systems to establish proper surveillance and evaluate the effectiveness of public health protocols tailored by risk severity.

Here, we aimed to develop a scale that can identify patients at risk of severe influenza outcomes, thus helping to guide preventive and therapeutic interventions.

## Methods

### Data source

The Canadian Immunization Research Network (CIRN) is a nationwide group of top vaccine experts working on vaccine safety, effectiveness, and acceptance (https://cirnetwork.ca/). They also focus on the implementation and evaluation of vaccination programs. CIRN plays a key role in providing research insights that help shape public health decisions related to vaccinations, ultimately benefiting the health of Canadians. The CIRN Serious Outcomes Surveillance (SOS) Network, established in 2009, aims to understand the impact of influenza and assess how effective seasonal flu vaccines are. As such, hospitalized patients meeting a broad definition of acute respiratory illness who have been tested for influenza are enrolled, either as test-positive influenza cases or test-negative controls. The SOS Network actively monitors influenza cases at multiple hospitals across several Canadian provinces each season^3,10–14^, gathering data from different sites depending on available resources. This study used pooled data from the CIRN SOS Network database.

### Participants

We used data from the 2011/2012 to 2018/2019 influenza seasons, selecting all patients with laboratory-confirmed influenza infection. The present analyses used data collected during the initial assessment of the patient within the hospital, which reflects the patient's condition immediately after being hospitalized. All hospitalized patients across the full range of illness severity were included in the present analyses, including those with and without supplemental oxygen and those requiring ventilatory support and ICU admission. There were no other data filters, and we kept all cases with missing values.

The study adhered to the guidelines outlined in the Declaration of Helsinki. The Research Ethics Boards approved the protocol, including data and sample collection and medical record review at all participating institutions (ClinicalTrials.gov Identifier: NCT1517191).

### Definition of influenza infection

Nasopharyngeal swab samples from all participating subjects underwent reverse transcription polymerase chain reaction (RT-PCR) influenza testing^15^. Subjects were classified as “laboratory-confirmed cases” if they tested positive for influenza or “negative controls” if they tested negative. Only laboratory-confirmed influenza cases were included in the present analyses.

### Data collection

Demographic and clinical data collection followed a standardized CIRN SOS Network protocol described elsewhere^13,16^. A broad set of variables from the SOS dataset were fed into model development. Demographic data included sex and age. Health-related data included: smoking status, clinical symptoms and signs (feverishness, nasal congestion, headache, abdominal pain, malaise, cough, diarrhea, weakness, shortness of breath, vomiting, dizziness, sore throat, nausea, muscle aches, arthralgia, prostration, seizures, myalgia, sneezing, conjunctivitis, sputum production, chest pain, encephalitis, nose bleed, altered consciousness, chills, and anorexia), function i.e. degree of dependence on activities of daily living (transferring, ambulating, need for assistive devices to ambulate, balance, bathing, toileting, handling medications, dressing, eating, handling finances), need for regular support for activities of daily living, need for additional support for activities of daily living, sensory disturbances (vision, hearing, and speech), bladder and bowel dysfunction, appetite disturbances, and comorbidities (ischemic heart disease, cardiac arrhythmias, valvular disease, congestive heart failure, hypertension, peripheral vascular disease, cerebrovascular disease, dementia, other noncognitive neurological disorders, hemiplegia/paraplegia, chronic pulmonary disease, pulmonary vascular disease, rheumatological disease, peptic ulcer disease, liver disease, diabetes mellitus, solid tumor, any type of metastatic cancer, HIV/AIDS, hypothyroidism, lymphoma, coagulopathy, blood loss anemia, deficiency anemia, alcohol abuse, drug abuse, obesity, involuntary weight loss, fluid and electrolyte disorders, edema, any psychiatric disease, depression, and peripheral skin ulcers). Influenza vaccination status was deemed “vaccinated” for those who received a current season flu vaccine more than 14 days before the onset of symptoms and “unvaccinated” if otherwise. Data collection was done by on-site study monitors who obtained the data for each patient based on the best possible source, including a review of patient charts or medical records and interviews with patients, family members, and healthcare team members where required. Influenza vaccination status was verified using medical records or registries where available or through contact with the immunizing health care professional.

### Outcomes of interest

The outcome of interest in this sample of patients was defined as the occurrence of a Major Clinical Event (MCE). We chose this outcome as it is a specific and measurable health event for which all study subjects were at risk at the time of hospitalization. MCE was defined as a composite outcome of the need for supplemental oxygen therapy, admission to an intermediate care unit, need for non-invasive or invasive ventilation, admission to an intensive care unit, or death. To assess the diagnostic accuracy of the scale in predicting MCE outcome, two steps were taken: (1) assessing the scale's overall diagnostic performance as a continuous variable and (2) grouping scores into three risk categories (low, moderate, and high) based on sensitivity and specificity values. The population's risk level must be considered when deciding on the test’s minimum sensitivity and specificity levels. For low-risk individuals, a minimum specificity of 50% and maximum sensitivity should be chosen to ensure that those at low risk are identified without reducing the detectability of those at higher risk. For high-risk individuals, a minimum sensitivity of 50% and maximum specificity should be selected to ensure that those at higher risk are detected without reducing the screening of those at lower risk. The remaining scores were classified as moderate risk.

### Development workflow

Supplementary material Fig. 1 describes the workflow scheme.

**Figure 1:**
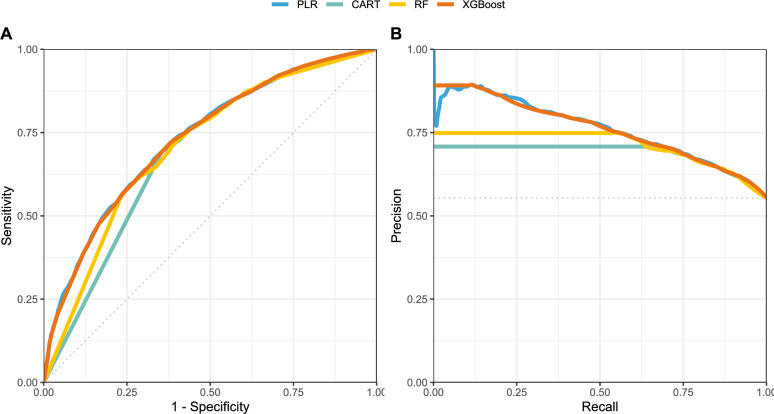
The (**A**) ROC and (**B**) PR curves of the models in predicting the occurrence of MCE on the test set.

#### Data preprocessing

We used a data-splitting approach to validate our findings. As per the studies conducted by Dobbin and Simon^17^ and Nguyen et al.^18^, a train-test splitting ratio of around 30% is considered reasonable. To provide an unbiased evaluation of the model fit on the training dataset while fine-tuning model hyperparameters, we also held out about 15% of the training set as a validation set. We randomly divided the total sample into three sets—a training set (60%), a validation set (10%), and a test set (30%), each stratified based on their MCE status to ensure an equal balance between groups. We then transformed the raw data into a valuable and efficient format: missing values were kept at an "Unknown" level, categorical variables were converted into dummy variables, and the continuous variable (age) was centralized and standardized. The variable imbalances between sets were evaluated using standardized mean differences and differences in proportion. A strict threshold of 0.05 was applied to indicate significant imbalances between the groups^19^.

#### Variable selection

We utilized the training and validation sets for variable selection. We modeled the outcome as a function of all predicting variables using Random Forest, which generated a list of importance rankings based on the Gini index. Next, we applied the Random Forest algorithm and tenfold cross-validation to calculate the Area Under the Receiver Operating Characteristic Curve (AU-ROC). We progressively included variables from the list, starting with the highest rank, until we reached a saturation threshold of AU-ROC variance ≤ 1% for two consecutive iterations.

#### Generating weighted scores

Logistic Regression was applied to generate weighted scores, modeling MCE as a function of the chosen variables. Each variable’s β coefficient was divided by the lowest β coefficient and rounded to the nearest value. The sum of scores for each category gave the total score normalized to a range of 0–100 for practicality, with 100 representing the highest risk for MCE and 0 denoting a zero risk.

### Model evaluation

We evaluated the weighted scores' ability to predict MCE via four methods: Penalized Logistic Regression (PLR), Classification and Regression Trees (CART), Random Forest (RF), and eXtreme Gradient Boosting (XGBoost). We employed a stratified ten-fold cross-validation for each predictive model to select the ideal hyperparameter combination using grid search, then trained each model individually.

#### Evaluation metrics

We developed Receiver Operating Characteristic (ROC), Precision-Recall (PR), and Gain curves for each prediction model to evaluate the performance of the four algorithms on the test dataset. We employed six traditional metrics (AU-ROC, Area Under the Precision-Recall Curve [AU-PRC], sensitivity, specificity, precision, and F1 score) to generate predicted classes. Then, we determined point-estimated metrics by cross-tabulating the observed and predicted classes. We selected the best model based on the best performance in the ROC and PR spaces^20^.

### Data analysis

All analyses were conducted using was performed in R (version 4.2.1) using RStudio IDE (RStudio 2022.02.1.461 “Prairie Trillium” Release).

### Ethics approval and participation consent

All participants provided informed consent for data, sample collection, and medical record screening per the local Research Ethics Boards' requirements. The Research Ethics Boards approved the protocol of participating institutions (ClinicalTrials.gov Identifier: NCT01517191).

## Results

The original dataset enrolled 24,068 participants; 12,954 (53.8%) had laboratory-confirmed influenza infections. Supplementary Table 1 presents the overall characteristics of the study population and indicates no significant differences (above the 0.05 threshold) among the three datasets (training, validation, and test sets).

### Variable selection and weighting

Supplementary Figs. 3 and 4 indicate that a list of importance rankings based on the Gini index was observed for the predicting variables and the cumulative ten-fold cross-validated AU-ROC estimates until reaching the saturation threshold for two consecutive iterations. This led us to select ten variables from four domains: demographic (age and sex), health history (smoking status, chronic pulmonary disease, diabetes mellitus, and influenza vaccination status), clinical presentation (cough, sputum production, and shortness of breath), and function (need for regular support for activities of daily living). Table 1 displays the resulting scale and its weighted scores. The scale displayed a right-skewed distribution (Supplementary Fig. 2A), with a median value of 39 across all participants (1st–3rd quartile, 23–59), and no differences between women and men (Supplementary Fig. 2B).

**Table 1:** The Influenza Severity Scale (ISS).

Domain	Parameter	Level	Score
Demographic	Age group	< 66 years	0
		≥ 66 years	5
	Sex	Male	0
		Female	2
Health history	Smoking status	Never smoked	0
		Former smoker	5
		Current smoker	7
		Unknown	9
	Chronic Pulmonary Diseases	No	0
		Yes	20
	Diabetes mellitus	No	0
		Yes	4
	Vaccination in the current influenza season	Yes	0
		No	1
Clinical presentation	Cough	No	0
		Yes	4
	Sputum production	No	0
		Yes	7
	Shortness of breath	No	0
		Yes	30
Function	Require regular support for activities of daily living	Unknown	0
		No	7
		Yes	18
Total score			100
Risk stratification			
Low risk			≤ 30
Moderate risk			> 30 and < 50
High risk			≥ 50

### Comparing performance across different models

Table 2 reveals similar accuracy of predictive models on the training set, with minor disparities in AU-ROC. We chose not to exclude any models before assessing their performance on the test set. Figure 1 displays the ROC and PR curves of the prediction models on the test set, with individual ROC and gain curves in the Supplementary material (Figs. 5–8). Results show that all models had acceptable discrimination performance, ranging from 69.2 to 73.1% in AU-ROC, similar to those in their training set. Examining the ROC and PR curves, the Penalized Logistic Regression and eXtreme Gradient Boosting models demonstrate the best overall performance when using the ISS as a continuous variable.

**Table 2:** Performance of the models in predicting the occurrence of MCE on the train and test sets.

Evaluation metric	Penalized Logistic Regression	Classification and regression trees	Random Forest	eXtreme Gradient Boosting
Train set				
AU-ROC*	0.730	0.690	0.709	0.728
Test set				
AU-ROC*	0.731	0.692	0.707	0.730
AU-PRC**	0.759	0.687	0.711	0.760
Accuracy	0.673	0.670	0.666	0.668
Specificity	0.563	0.562	0.527	0.522
Sensitivity	0.762	0.758	0.778	0.785
Precision	0.684	0.682	0.671	0.671
F1 score	0.721	0.718	0.721	0.723

*AU-ROC, Area Under the Receiver Operating Characteristic curve.

**AU-PRC, Area Under the Precision-Recall curve.

### Finding the optimal cutoff points

**Figure 2:**
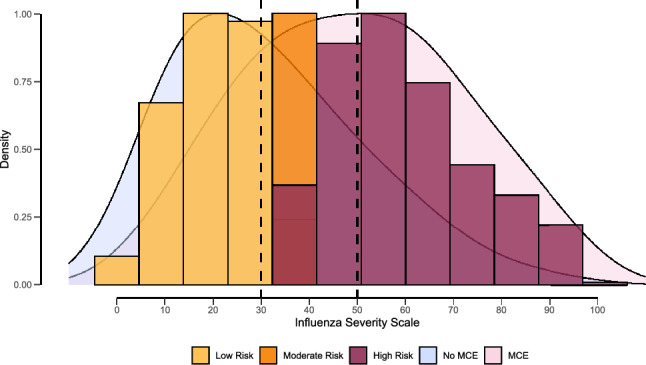
The histogram of the ISS scores by risk categories overlaps a density plot by the outcome MCE. Sturges’ Rule determined the optimal number of bins.

When tested on the set, the ISS attained a remarkable AU-ROC of 0.73 (95% CI, 0.71–0.74). When applying the Youden index to determine the optimal score threshold, it revealed a value of ≥ 37, leading to a sensitivity of 0.70 (95% CI, 0.68–0.72) and a specificity of 0.63 (95% CI, 0.60–0.65). To interpret the scores simpler, Supplementary Table 2 provides a conversion table that maps the cutoff values to their respective predicted risks and performance metrics. For further simplification, the scores were aggregated into three categories: low risk (ISS < 30; sensitivity 79.9% [95% CI, 78–81.7%], specificity 51% [95% CI, 48.6–53.3%]), moderate risk (ISS ≥ 30 but < 50; 54.5% sensitivity, 55.9% specificity) and high risk (ISS ≥ 50; sensitivity 51.4% [95% CI, 49.3–53.6%], specificity 80.5% [95% CI, 78.7–82.4%]; Fig. 2).

## Discussion

This study demonstrates the development and assessment of accuracy for the Influenza Severity Scale. This 10-item scale was designed to differentiate patients infected with influenza by their risk of experiencing major clinical events. The ISS is based on simple, easily accessible data and discriminates accurately. This emphasizes the utility of the ISS for both retrospective and prospective studies in accurately assessing and predicting influenza-related outcomes.

Adams and colleagues reviewed common severity assessments for influenza and community-acquired pneumonia^7^. They examined 118 studies focusing on influenza, which included evaluations of tools such as PSI, CURB 65, APACHE II, SOFA, and qSOFA^21–35^. The clinical outcomes studied in these assessments involved mortality rates (overall, in ICU, in hospital), ICU admissions, mechanical ventilation needs, length of hospital stays, and total hospitalizations. Despite the extensive findings, none of the assessments were specifically designed for influenza or considered other critical outcomes like admission to intermediate care units or the need for oxygen therapy or non-invasive ventilation. Additionally, significant differences were observed in the clinical parameters and research endpoints across the studies reviewed. These variations made it challenging to combine results and perform a meta-analysis accurately. Consequently, it hindered the authors from making precise evaluations of diagnostic performance and conducting direct comparisons between different assessment tools.

Nevertheless, although PSI and CURB-65 are generally reliable in predicting 30-day mortality rates for community-acquired pneumonia in different clinical settings, some studies suggest that they may not be effective in predicting mortality rates for influenza pneumonia cases. For example, in a study conducted by Riquelme et al.^36^, these pneumonia scoring systems were ineffective in predicting the survival rate of low-risk patients with the H1N1 2009 influenza pandemic. Another study revealed that these scoring systems are still inefficient for the influenza pandemic because they cannot accurately predict the need for intensive care services^37^. The study revealed promising findings for SMRT-CO in identifying low-risk patients, with an AU-ROC of 0.826^37^.

In contrast, other studies found that the AU-ROC values for predicting mortality in patients with influenza A were 0.777 for CURB-65 and 0.560 for PSI^22^. This study proposed the FluA-p score as a novel approach to predict mortality in patients with influenza A-related pneumonia, achieving an AU-ROC of 0.908^22^. However, despite its high AU-ROC, the FluA-p score relies on laboratory variables as risk parameters, similar to the SOFA score. This reliance may present challenges when incorporating it into environments with limited resources or assessing severity in epidemiological studies that often lack access to laboratory data.

When analyzing medical research data with categorical outcomes, it's crucial to consider performance metrics. While the ROC curve is commonly used to assess test performance, dealing with imbalanced datasets can distort results. Combining ROC and Precision-Recall (PR) curves along with their respective AUC measurements (AU ROC and AU PR) is recommended to address this issue. Surprisingly, PR curves are often overlooked in diagnostic performance studies in influenza patients. To fill this gap, we assessed ISS's discriminative ability using ROC and PR curves. The ISS demonstrated strong discriminative performance in the test set, achieving AU ROC and AU PR values exceeding 70%. Remarkably, these results were achieved without using any laboratory parameters and by including patients from various sites to minimize bias stemming from local practices.

Estimating disease severity involves assessing the probability of significant clinical events among those individuals who are at risk but have not yet experienced any at the start of the observation period. This probability is determined by a set of parameters, some of which can be modified while others cannot. The severity assessment must consider modifiable and non-modifiable parameters, with the latter being the most important. Modifiable parameters may not always lead to a decrease in the risk of clinical events, but they can indicate how an intervention, disease progression, or host response will affect the baseline risk. Even if the modifiable parameters do not indicate a high risk, this does not necessarily mean the individual's baseline risk has changed. Instead, it reflects the natural history of the disease and how it interacts with the host. Therefore, individuals must be stratified based on modifiable and non-modifiable risks. Our tool considers this, allowing us to gain insight into the progression of the disease and the host's response to the infection. At the same time, it recognizes and respects the individual's particularities and dynamic responses.

To ensure the most effective risk management strategies, ISS utilizes a 3-threshold system to differentiate between low- and high-risk patients when measuring the likelihood of major clinical events. These thresholds were specifically chosen to ensure a high sensitivity and specificity rate exceeding 80%. This 3-tier system facilitates risk-based treatment protocols and permits a more centralized and cost-effective care distribution. For medical personnel, this allows for treatments and services to be tailored to the specific needs of patients, enabling higher-quality care and more successful patient outcomes. During epidemiological studies, the use of ISS enables the identification of the disease severity and its association with a particular strain. It can also be used as an adjustment measure when assessing the effectiveness of interventions.

Our study has several limitations that must be considered. Firstly, the data we used was obtained from hospital surveillance, which inherently limits our ability to account for specific institutional protocols. Our data consisted of the initial assessment of the patient within the hospital, which reflects the patient’s condition immediately after being hospitalized. While using this approach, we might have lost the ability to capture the scale’s performance in predicting the need for hospitalization. However, we could still track all significant clinical events that occurred afterward. Also, we have not had lower tract samples, meaning we may not have identified some people with severe disease, thus skewing the results. Moreover, our tool's reliance on non-modifiable parameters limits its capacity to be used as an evolutionary or responsive variable during hospitalization, as it does not concentrate on physiological parameters. Unsurprisingly, the absence of modifiable parameters, such as vital signs, laboratory, and radiological variables, impacted the discriminatory performance of our tool. Future studies should include these variables to enhance its performance while ensuring a balance with non-modifiable parameters. Some rare characteristics are likely underrepresented in our cohort, leading to an insufficient data sample to identify them as significant factors influencing ISS. Nonetheless, they could be relevant in other settings, also warranting further validation. Lastly, ISS comprises ten variables and individual scores, which can be challenging to remember in clinical practice. Ideally, future work could focus on developing automated means of collecting and calculating the ISS to support its use in research and clinical settings. Despite these drawbacks, our dataset was sizeable, multi-centric, and included a wide variety of people who are generally not included in these kinds of studies, such as individuals on the extremes of age with or without comorbidities.

In summary, the ISS is a tool used to assess the severity of influenza infection. It considers the patient's symptoms and non-modifiable parameters to predict the likelihood of MCE without requiring lab tests other than confirming influenza infection. This can help direct protocols and policies to those at greater risk of MCE, such as older adults, women, smokers, CPD patients, diabetics, and those not vaccinated against influenza. It also highlights the importance of considering multiple factors (and their intersection) contributing to a person's risk rather than individual factors considered singly.

Additionally, the tool can raise awareness of *important symptoms* that predict worse outcomes, namely (in order of importance) shortness of breath, sputum production, and coughing, as well as *important clinical features* such as underlying health conditions and functional status. Notably, the most important clinical factors identified here were underlying chronic lung disease, shortness of breath, and baseline functional impairment with the requirement for support in activities of daily living. These are relevant and readily identifiable factors that impact clinical prognostication and decision-making. For example, a 70-year-old patient with baseline functional impairment who presents with shortness of breath is at high risk for MCE from influenza, and this can be communicated to the patient and family early in their admission.

We believe ISS will be essential for public health systems to monitor the effects of public health protocols on clinical outcomes and establish efficient surveillance measures. Further research is necessary to explore the utility of ISS in different clinical settings and its capacity to predict mortality. Ultimately, the ISS may prove to be a valuable metric for assessing and improving influenza-related health outcomes, contributing to the betterment of public health.

### Supplementary Information

Supplementary Information.

## Data Availability

The datasets generated and/or analysed during the current study are not publically available due to the confidential nature of the data obtained from patients, however, datasets are available through the corresponding author on reasonable request.
