# Supplementary material for: Predicting major clinical events among Canadian adults with laboratory-confirmed influenza infection using the influenza severity scale
Source: Sci Rep. 2024 Aug 8;14:18378. doi: 10.1038/s41598-024-67931-9 (PMC11306731; doi:10.1038/s41598-024-67931-9)
Supplement: Supplementary file 1 — Supplementary Information. [file 41598_2024_67931_MOESM1_ESM.docx]

**Supplementary material**

The following content is supplementary material for the study "**Influenza Severity Scale (ISS): Predicting major clinical events among Canadian adults with laboratory-confirmed influenza infection**." This supplementary material includes additional data, figures, and detailed information that provide a more comprehensive view of the research conducted in the main study.

Table of Contents

[Figure 1. Scale development workflow scheme. 2](#_Toc166832380)

[Table 1. The characteristics of the train, test, and validation set populations. 3](#_Toc166832381)

[Figure 2. (A) Overall distribution of the Influenza Severity Scale scores and by sex (B); the solid line represents the fitted density plot of the scores. 4](#_Toc166832382)

[Figure 3. A Random Forest model generated a list of importance rankings based on the Gini index observed for the predicting variables. 5](#_Toc166832383)

[Figure 4. 10-fold cross-validation cumulatively estimating the AU-ROC by iterating over the input variables. The blue line represents the highest rank until reaching a saturation threshold for two consecutive iterations. 6](#_Toc166832384)

[Figure 5. (A) ROC and (B) gain curves for the Penalized Logistic Regression (PLR) model on the train set. 7](#_Toc166832385)

[Figure 6. (A) ROC and (B) gain curve for the Classification and Regression Trees (CART) model. 8](#_Toc166832386)

[Figure 7. (A) ROC and (B) gain curve for the Random Forest (RF) model. 9](#_Toc166832387)

[Figure 8. (A) ROC and (B) gain curve for the eXtreme Gradient Boosting (XGBoost) model. 10](#_Toc166832388)

[Table 2. Conversion table mapping pre-specified score cutoffs to their predicted risks. 11](#_Toc166832389)

[Table 3. The sociodemographic and clinical characteristics of the entire study population. 12](#_Toc166832390)

# Figure 1. Scale development workflow scheme.

**
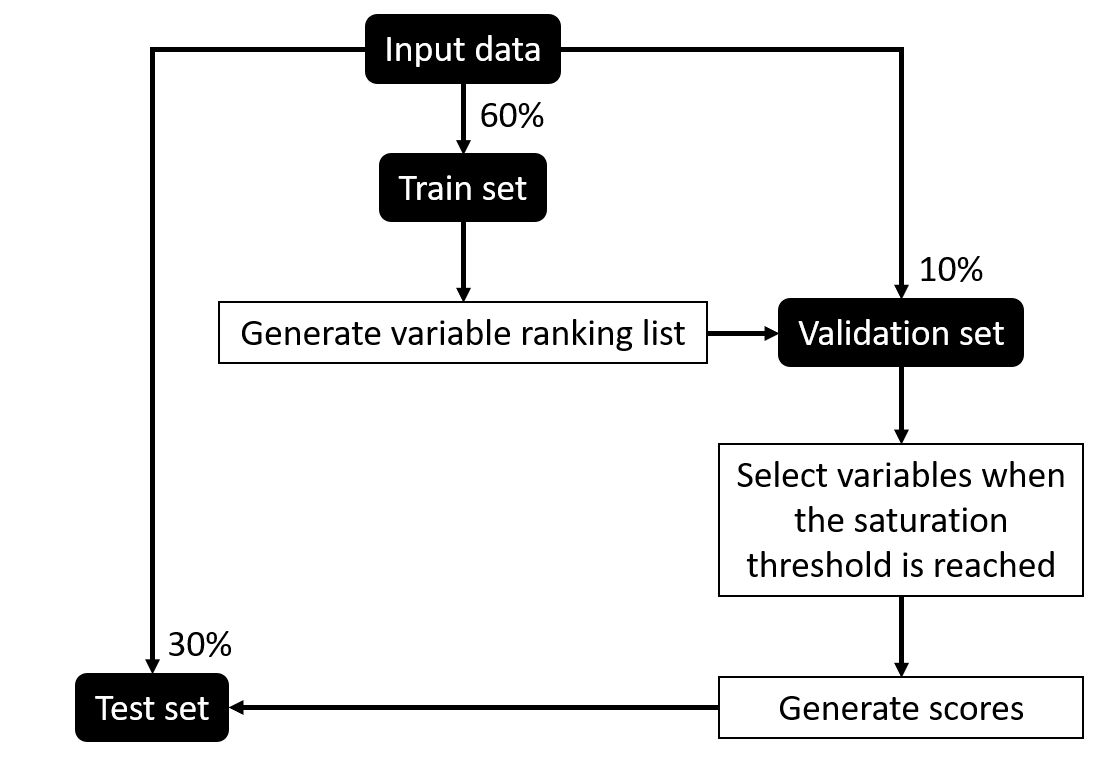
**

# Table 1. The characteristics of the train, test, and validation set populations.

| **Parameter** | **Level** | **Train set** | **Test set** | **Validation set** | **Balance by dataset pair: Standard difference*** | | |
| --- | --- | --- | --- | --- | --- | --- | --- |
|  |  | **(N=7,773)** | **(N=3,886)** | **(N=1,295)** | **Test vs. Train** | **Test vs. Validation** | **Train vs. Validation** |
| Age | Years | 74 [59 84] | 74 [59, 85] | 75 [61, 86] | -0.0190 | 0.0237 | 0.0427 |
| Sex | Female | 4163 (53.6) | 2110 (54.3) | 698 (53.9) | 0.0074 | 0.0040 | -0.0034 |
|  | Male | 3610 (46.4) | 1776 (45.7) | 597 (46.1) |  |  |  |
| Health history | Current smoker | 2045 (26.3) | 1034 (26.6) | 348 (26.9) | -0.0030 | 0.0026 | 0.0056 |
|  | Former smoker | 2255 (29.0) | 1077 (27.7) | 370 (28.6) | 0.0130 | 0.0086 | -0.0044 |
|  | Never smoked | 2940 (37.8) | 1479 (38.1) | 498 (38.5) | -0.0024 | 0.0040 | 0.0063 |
|  | Smoking status unknown | 533 (6.9) | 296 (7.6) | 79 (6.1) | -0.0076 | -0.0152 | -0.0076 |
|  | Chronic Pulmonary Diseases | 1846 (23.7) | 891 (22.9) | 265 (20.5) | 0.0082 | -0.0247 | -0.0329 |
|  | Diabetes mellitus | 2143 (27.6) | 1070 (27.5) | 341 (26.3) | 0.0004 | -0.0120 | -0.0124 |
|  | Vaccination in the current influenza season | 2849 (36.7) | 1382 (35.6) | 463 (35.8) | 0.0109 | 0.0019 | -0.0090 |
| Clinical presentation | Cough | 5814 (74.8) | 2939 (75.6) | 955 (73.7) | -0.0083 | -0.0189 | -0.0105 |
|  | Sputum production | 2918 (37.5) | 1549 (39.9) | 507 (39.2) | -0.0232 | -0.0071 | 0.0161 |
|  | Shortness of breath | 2817 (36.2) | 1388 (35.7) | 440 (34.0) | 0.0052 | -0.0174 | -0.0226 |
| Function | Require regular support for activities of daily living | 3490 (44.9) | 1722 (44.3) | 595 (45.9) | 0.0072 | 0.0117 | 0.0045 |
| Outcomes | Major Clinical Events | 4508 (58.0) | 2151 (55.4) | 744 (57.5) | 0.0264 | 0.0210 | -0.0054 |

* Standardized mean difference (for continuous variables) and difference in proportions (for categorical variables).

# Figure 2. (A) Overall distribution of the Influenza Severity Scale scores and by sex (B); the solid line represents the fitted density plot of the scores.


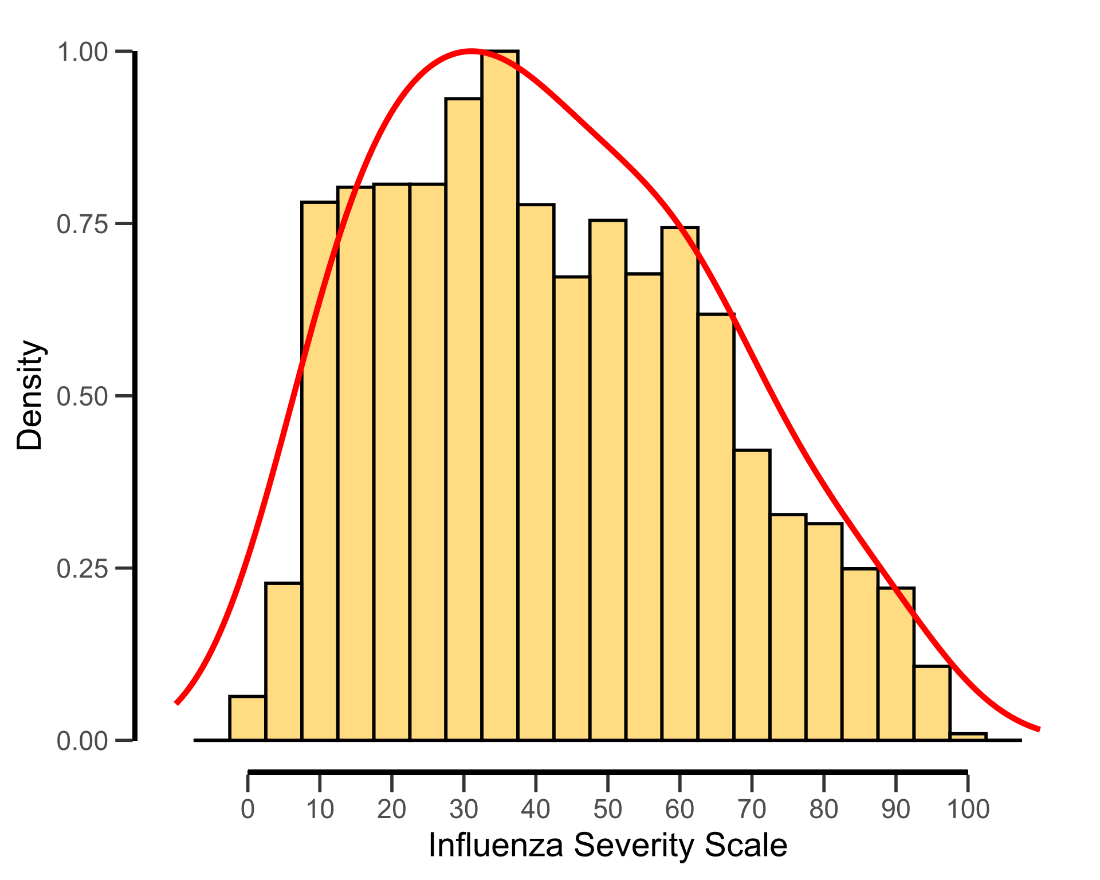


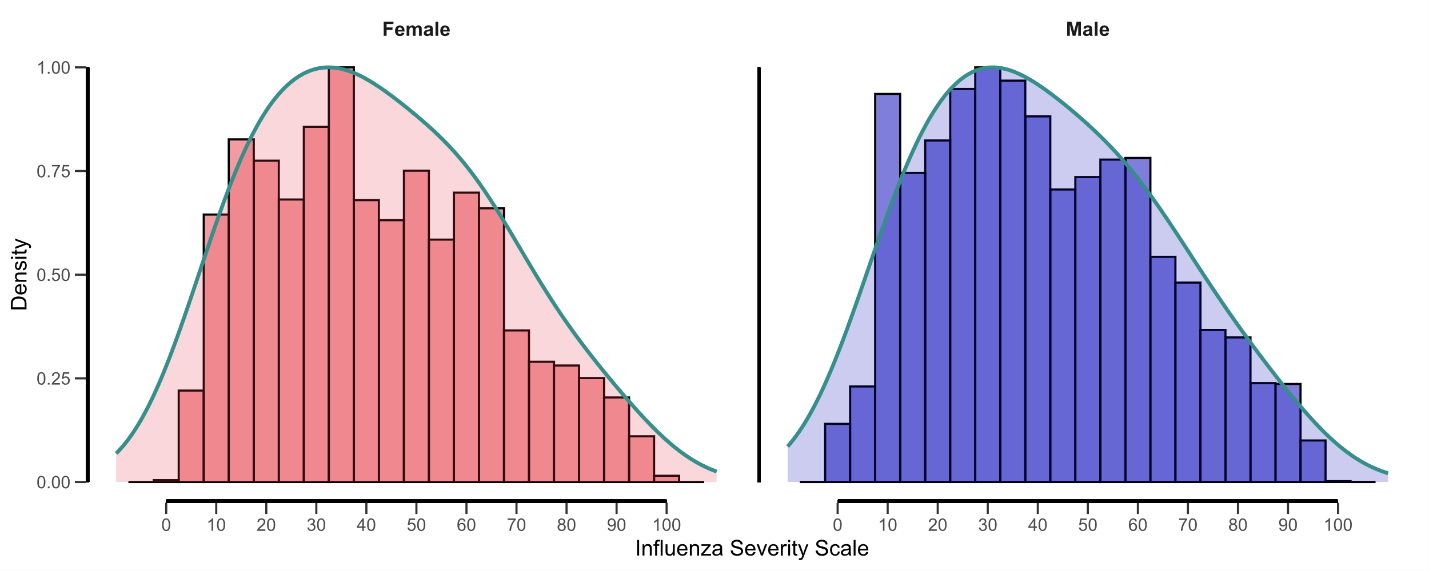


# Figure 3. A Random Forest model generated a list of importance rankings based on the Gini index observed for the predicting variables.


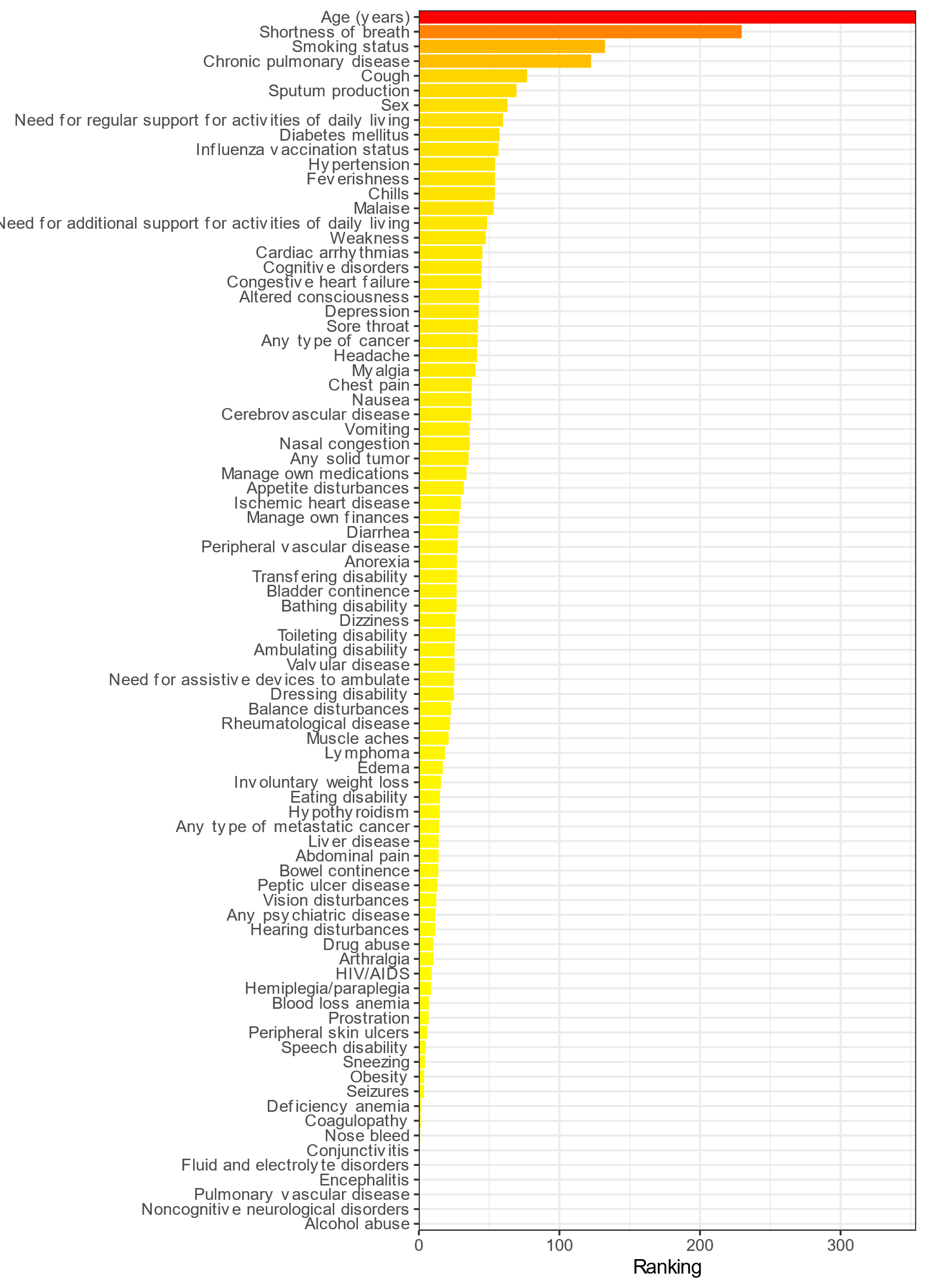


# Figure 4. 10-fold cross-validation cumulatively estimating the AU-ROC by iterating over the input variables. The blue line represents the highest rank until reaching a saturation threshold for two consecutive iterations.


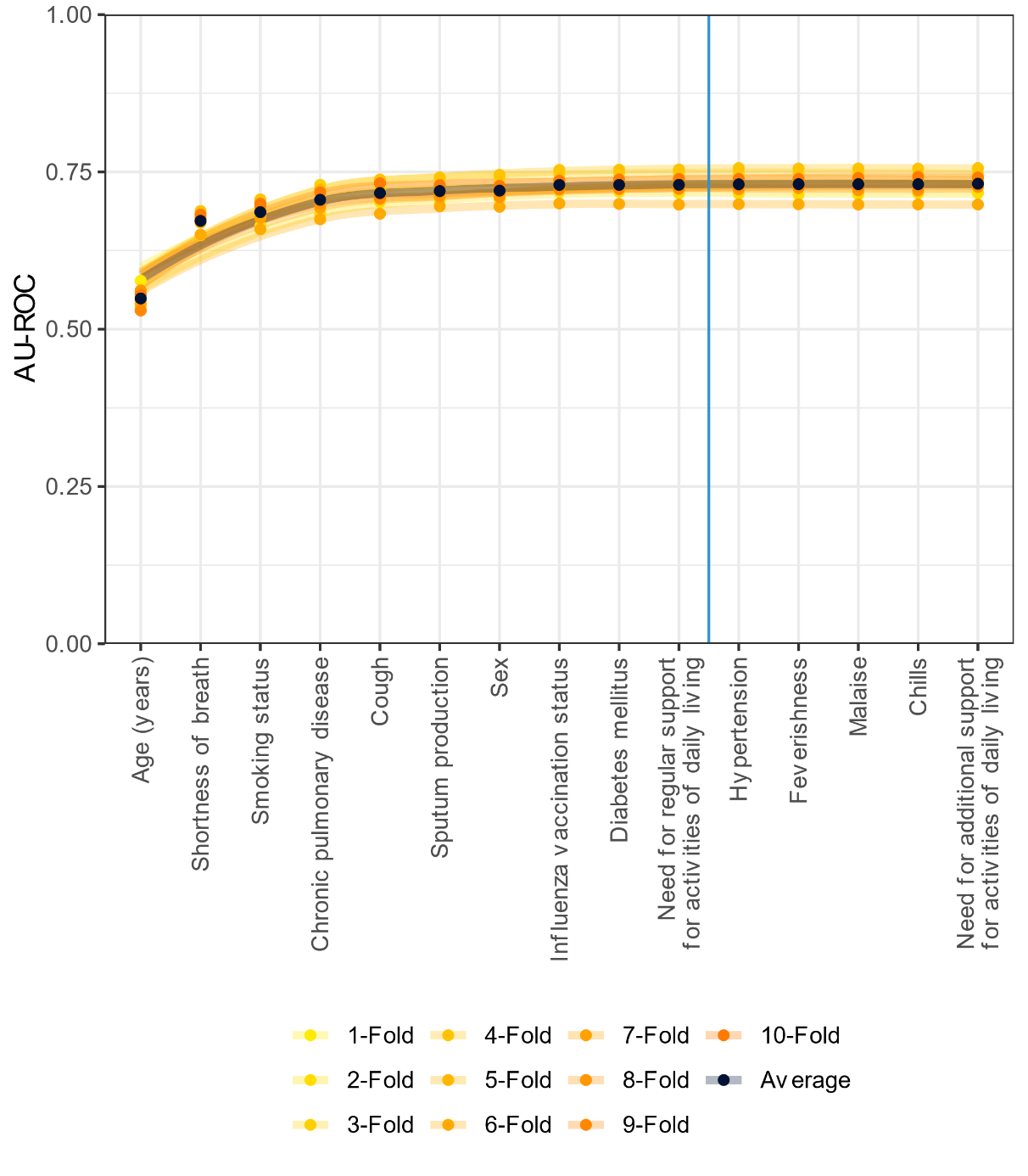


# Figure 5. (A) ROC and (B) gain curves for the Penalized Logistic Regression (PLR) model on the train set.


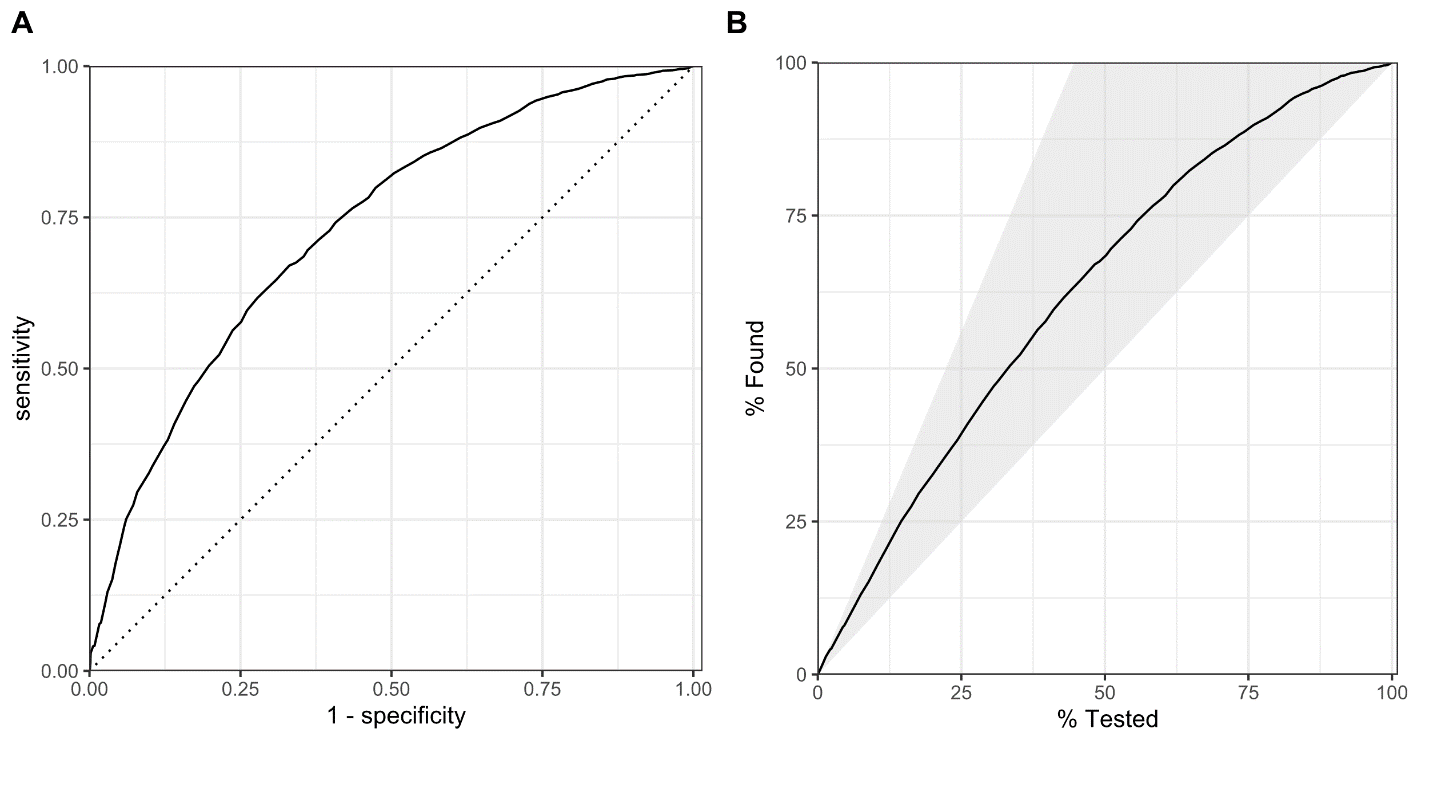


# Figure 6. (A) ROC and (B) gain curve for the Classification and Regression Trees (CART) model.


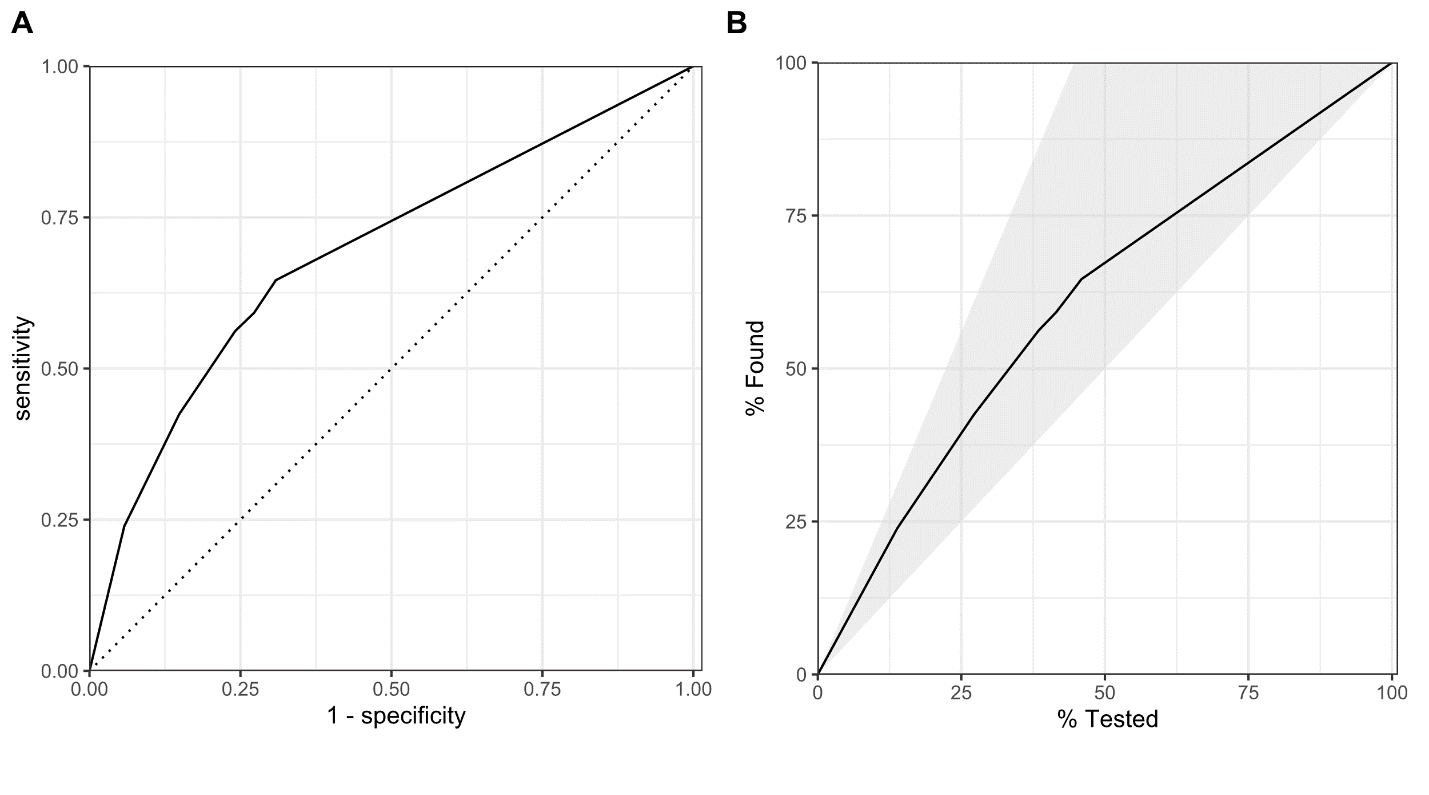


# Figure 7. (A) ROC and (B) gain curve for the Random Forest (RF) model.


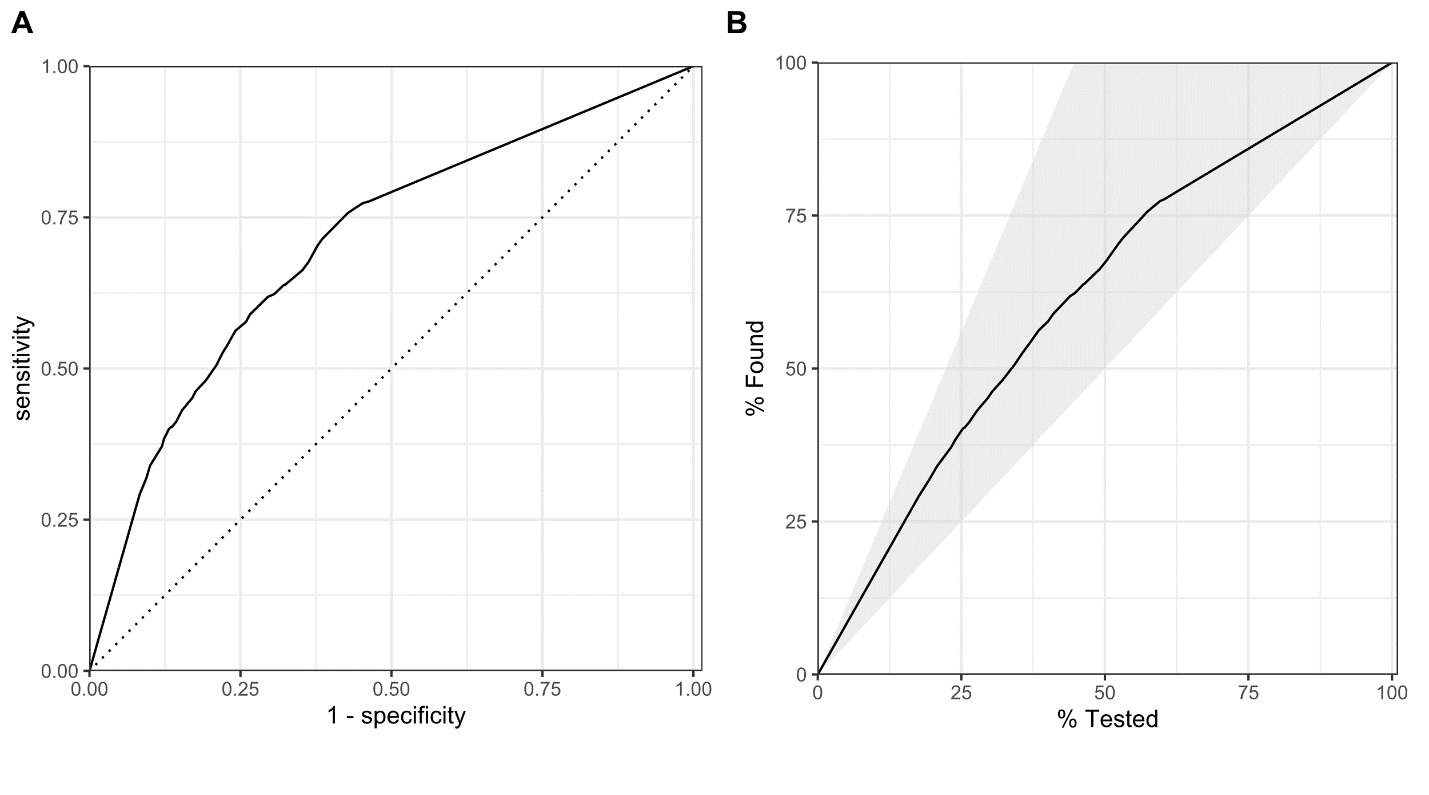


# Figure 8. (A) ROC and (B) gain curve for the eXtreme Gradient Boosting (XGBoost) model.


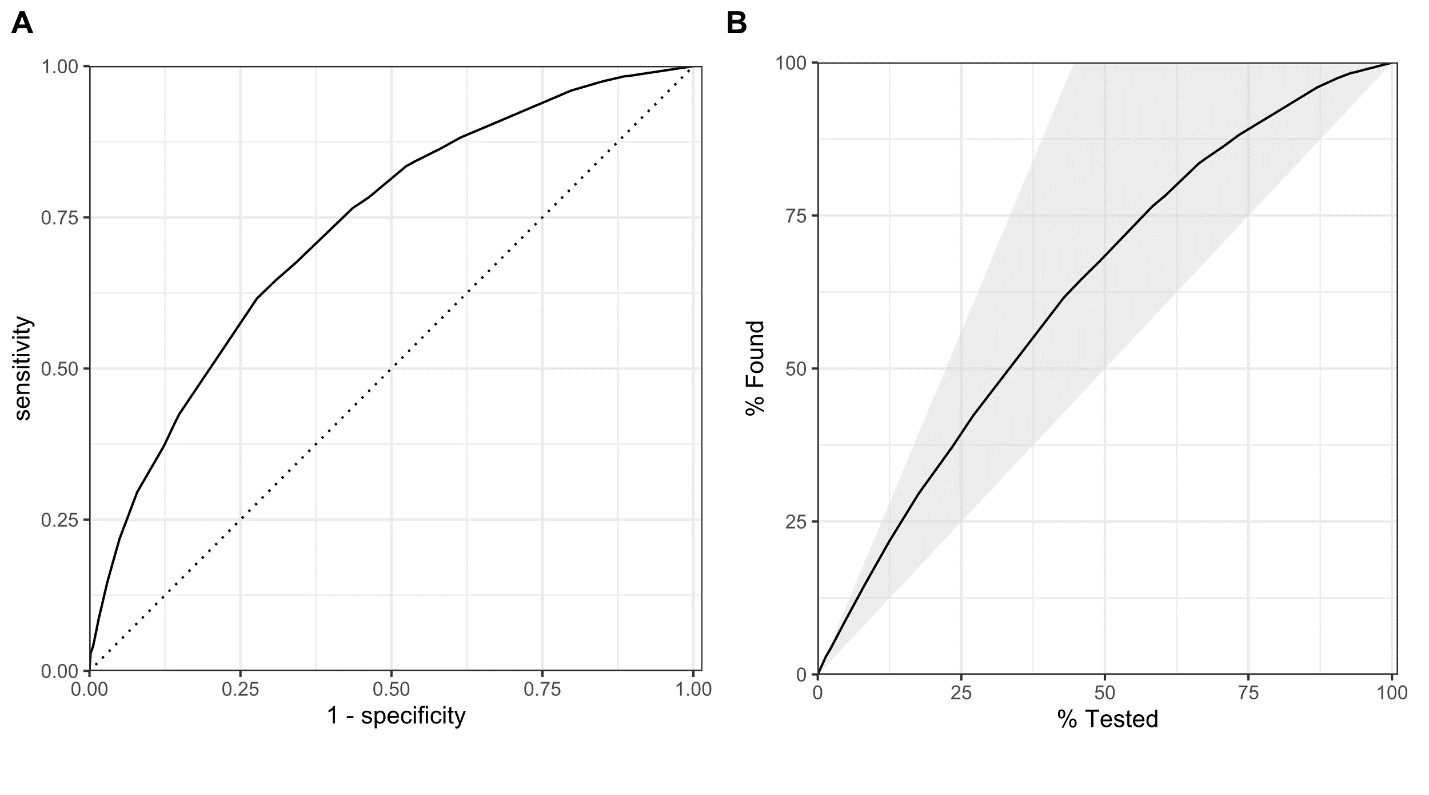


# Table 2. Conversion table mapping pre-specified score cutoffs to their predicted risks.

| **Score**  **cutoff (≥)** | **Predicted risk (≥)** | **Percentage of patients (%)** | **Sensitivity**  **(95% CI)** | **Specificity**  **(95% CI)** | **Risk classification** |
| --- | --- | --- | --- | --- | --- |
| 18 | 33.10% | 84 | 92.9% (91.8-94.0%) | 26.7% (24.7-28.8%) | **Low risk** |
| 20 | 34.90% | 80 | 90.6% (89.3-91.8%) | 32.3% (30.1-34.5%) |  |
| 22 | 36.80% | 77 | 88.2% (86.8-89.6%) | 36.6% (34.3-38.8%) |  |
| 24 | 38.60% | 75 | 86.6% (85.0-88.0%) | 39.8% (37.5-42.1%) |  |
| 26 | 40.50% | 72 | 84.8% (83.4-86.3%) | 43.7% (41.6-46.2%) |  |
| 28 | 42.50% | 69 | 82.4% (80.8-84.1%) | 47.2% (45.0-49.6%) |  |
| 30 | 44.50% | 66 | 79.9% (78.0-81.7%) | 51.0% (48.6-53.3%) |  |
| 32 | 46.40% | 62 | 76.5% (74.7-78.2%) | 55.9% (53.7-58.3%) | **Moderate risk** |
| 34 | 48.40% | 60 | 74.8% (72.9-76.7%) | 57.8% (55.4-60.1%) |  |
| 36 | 50.40% | 57 | 72.5% (70.6-74.3%) | 61.1% (58.8-63.3%) |  |
| 38 | 52.40% | 54 | 69.3% (67.3-71.1%) | 64.3% (62.1-66.6%) |  |
| 40 | 54.40% | 51 | 66.1% (64.1-68.1%) | 67.3% (65.1-69.4%) |  |
| 42 | 56.40% | 49 | 63.5% (61.5-65.6%) | 69.2% (67.1-71.4%) |  |
| 44 | 58.40% | 45 | 59.8% (57.7-61.8%) | 73.3% (71.3-75.3%) |  |
| 46 | 60.30% | 42 | 57.3% (55.1-59.4%) | 76.0% (74.1-78.0%) |  |
| 48 | 62.20% | 40 | 54.5% (52.4-56.5%) | 77.7% (75.7-79.6%) |  |
| 50 | 64.00% | 37 | 51.4% (49.3-53.6%) | 80.5% (78.7-82.4%) | **High risk** |
| 52 | 65.90% | 34 | 48.0% (46.0-50.2%) | 83.3% (81.6-85.0%) |  |
| 54 | 67.60% | 32 | 45.7% (43.7-47.8%) | 84.6% (82.8-86.3%) |  |
| 56 | 69.40% | 30 | 42.9% (40.8-45.0%) | 85.8% (84.1-87.4%) |  |
| 58 | 71.00% | 28 | 39.7% (37.7-41.8%) | 87.4% (85.9-89.0%) |  |

# Table 3. The sociodemographic and clinical characteristics of the entire study population.

| **Parameter** | **Level** | **Study population** |
| --- | --- | --- |
|  |  | **(N=12,954)** |
| Age | Years | 74 [59, 85] |
| Sex | Female | 6,971 (53.8) |
|  | Male | 5,983 (46.2) |
| Health history | Smoking status |  |
|  | Current smoker | 3,427 (26.5) |
|  | Former smoker | 3,702 (28.6) |
|  | Never smoked | 4,917 (38.0) |
|  | Smoking status unknown | 908 (7.0) |
|  | Comorbidities |  |
|  | Chronic Pulmonary Diseases | 3,002 (23.2) |
|  | Pulmonary circulation disorders | 0 (0) |
|  | Diabetes mellitus | 3,554 (27.4) |
|  | Hypothyroidism | 339 (2.6) |
|  | Hypertension | 3,673 (28.4) |
|  | Myocardial infarction | 1,175 (9.1) |
|  | Congestive heart failure | 1,485 (11.5) |
|  | Cardiac arrhythmias | 2,534 (19.6) |
|  | Valvular disease | 771 (6.0) |
|  | Peripheral vascular disease | 868 (6.7) |
|  | Cerebrovascular disease | 1,505 (11.6) |
|  | Rheumatoid disease | 481 (3.7) |
|  | Peptic ulcer disease | 227 (1.8) |
|  | Cancer (any malignancy) | 2,409 (18.6) |
|  | Solid tumour, without metastasis | 1,630 (12.6) |
|  | Metastatic solid tumour | 278 (2.1) |
|  | AIDS/HIV | 77 (0.6) |
|  | Drug abuse | 186 (1.4) |
|  | Psychoses | 166 (1.3) |
|  | Depression | 1,244 (9.6) |
|  | Vaccination in the current influenza season |  |
|  | Unvaccinated | 8,260 (63.8) |
|  | Vaccinated | 4,694 (36.2) |
| Clinical presentation | Feverishness | 3,022 (23.3) |
|  | Nasal congestion | 1,313 (10.1) |
|  | Headache | 1,435 (11.1) |
|  | Abdominal pain | 321 (2.5) |
|  | Malaise | 2,622 (20.2) |
|  | Cough | 9,708 (74.9) |
|  | Diarrhea | 857 (6.6) |
|  | Weakness | 2,619 (20.2) |
|  | Shortness of breath | 4,645 (35.9) |
|  | Vomiting | 1,358 (10.5) |
|  | Dizziness | 702 (5.4) |
|  | Sore throat | 1,591 (12.3) |
|  | Nausea | 1,360 (10.5) |
|  | Muscle aches | 605 (4.7) |
|  | Arthralgia | 203 (1.6) |
|  | Prostration | 118 (0.9) |
|  | Seizures | 41 (0.3) |
|  | Myalgia | 1,630 (12.6) |
|  | Sneezing | 70 (0.5) |
|  | Conjunctivitis | 20 (0.2) |
|  | Sputum production | 4,974 (38.4) |
|  | Chest pain | 1,230 (9.5) |
|  | Encephalitis | 5 (0.0) |
|  | Nose bleed | 27 (0.2) |
|  | Altered consciousness | 1,636 (12.6) |
|  | Chills | 2,719 (21.0) |
|  | Anorexia | 999 (7.7) |
| Support for activities of daily living | Require regular support | 5,807 (44.8) |
|  | Need for additional support | 2,325 (17.9) |
| Outcomes | Major Clinical Events | 7,403 (57.1) |
|  | Supplemental oxygen therapy | 6,705 (51.8) |
|  | Non-invasive ventilation | 882 (6.8) |
|  | Invasive ventilation | 921 (7.1) |
|  | Intermediate Care Unit | 404 (3.1) |
|  | Intensive Care Unit | 1,805 (13.9) |
|  | Death | 916 (7.1) |
